# Parliament and Publications

**Published:** 1910-10-22

**Authors:** 


### PARLIAMENT AND PUBLICATIONS.

### HOSPITALS IN CEYLON.

Some interesting particulars concerning the hospitals of Ceylon are contained in a Government publication dealing with that colony, which has just been issued. There are 75 Government hospitals fully equipped with the most modern hospital furniture and surgical appliances. The number of beds in the hospitals ranges from 30 to 425. In addition to the hospitals there are 588 Government dispensaries, and 220 estate dispensaries supported by the planters, who get their drugs free, up to the value of 50 cents a coolie. The lunatic asylum is situated in Colombo, and is the only one in the island. There are 586 inmates. The leper asylum has 345 patients. In association with Government, a home for incurables (containing 80 beds) is worked by a committee of Government officials and representatives of the public. "Colonial Reports. Ceylon." No. 653. Price 5½d.

### HOSPITALS IN THE STRAITS SETTLEMENTS.

The number of patients treated in the hospitals of the Federated Malay States last year was 73,192, as against 84,105 in the previous year. The death rate in the hospitals was 9.88 per cent. The number of out-patients treated was 150,086; 25,675 patients were treated in Perak by the travelling dispensaries. The report of the Acting President-General states that investigations have enabled the Government to control with some success the very prevalent disease of beri-beri. The admission of beri-beri cases amounted last year to 5,469, with a death-rate of 12.05 per cent.; as compared with 9,790 cases, and a death-rate of 19.52 in 1908. In all Government institutions the use of white rice is being discontinued, and cured or parboiled rice is being substituted, with very satisfactory results. "Straits Settlements" (Cd. 5373). Price 11½d.

